# Relationship between the gingival biotype and the results of root covering surgical procedures: A systematic review

**DOI:** 10.4317/jced.59783

**Published:** 2022-09-01

**Authors:** Brenda Y. Herrera-Serna, Olga P. López-Soto, Tatiana Chacón, Ana M. Montoya-Gómez, Daniela Agudelo-Flórez, Oscar H. Zuluaga-López

**Affiliations:** 1 PhD Health Sciences. Department of Oral Health. Universidad Autónoma de Manizales; 2 Master Clinical Epidemiology. Department of Oral Health. Universidad Autónoma de Manizales; 3 Specialist Periodontology and implantology. Department of Oral Health. Universidad Autónoma de Manizales; 4 Specialist Oral Rehabilitation. Department of Oral Health. Universidad Autónoma de Manizales

## Abstract

**Background:**

Tissue biotypes are related to the results of periodontal therapy, conventional prosthodontics, implant therapy and root covering procedures. We conducted a systematic review (SR) of the literature about the relationship between the gingival biotype and the results of root covering surgical procedures.

**Material and Methods:**

A PICO question was defined. Two independent reviewers conducted electronic and manual literature searches, which covered studies up to August 31, 2020, in the National Library of Medicine (PubMed MEDLINE), EBSCO, Science Direct and Cochrane. The inclusion criteria were all root covering procedures in individuals with different gingival biotypes. The final result of the different surgical techniques was evaluated by the Root Coverage Esthetic Score (RES). The Methodological Index for the identification of biases for Non-Randomized Studies was applied.

**Results:**

Only four articles fulfilled the inclusion criteria. These studies adequately reported the percentage of root coverage after surgery, and two recorded the entire RES index. Complete root coverage was achieved in a range between 70 - 86.12% in patients with thin biotype and between 77.8 - 96% in patients with a thick biotype. The aesthetic results were not compromised by the initial classification of the biotype. The treatments used were subepithelial connective tissue graft with coronally displaced flap or its modification. No significant differences were found.

**Conclusions:**

When the gingival biotypes are analyzed as independent variables, none of the root coverage procedures are affected by the classification of the gingival biotype.

** Key words:**Root coverage, gingival recession, gingival biotype.

## Introduction

An adequate diagnosis of the gingival biotype in clinical practice is considered essential for decision-making in cosmetic dentistry and implantology. Tissue biotypes are related to the results of periodontal therapy, conventional prosthodontics, implant therapy and root covering procedures.

The gingival biotype refers to the quality of the soft tissue that surrounds the tooth (1) . Thick biotypes are characterized by flat soft tissue, marked bone architecture, dense soft tissue, and highly fibrous soft tissue, with a greater extent of keratinized tissue (2). Thin biotypes are delicate, highly scalloped, and translucent in appearance. The soft tissue appears friable, with a minimal amount of keratinized tissue and a thin vestibular bone Table with possible presence of dehiscences and fenestrations. Thin biotypes are considered risky because they have been associated with gingival recessions after surgical or restorative treatment (2).

Gingival recession is defined as the migration of the marginal tissue toward the apical of the amelocement junction and may be related to aesthetic problems, dentinal hypersensitivity and carious lesions (3). Many surgical procedures have been proposed for gingival recession treatment. Langer and Langer (4) proposed the use of palatal subepithelial connective tissue grafts with predicTable results. Since then, various modifications have been implemented using different techniques for flap management, such as the techniques reported by Nelson (5) (a subpedicular connective tissue graft), Bruno (6) (a procedure without vertical incisions), and Harris (17) (a split-thickness double pedicle with graft). Subepithelial connective tissue grafting has shown high rates of root coverage and is associated with a good prognosis (8). The influence of flap thickness and gingival biotype on periodontal surgical techniques and its relationship with the clinical outcome of root coverage procedures have been little discussed in the literature (9). Recent evidence seems to indicate that there is tissue stability after surgery when the thickness is greater than 1.44 mm (10).

Adequate soft tissue coverage in the gingival area is one of the main concerns in the outcome of periodontal surgery and in restorative and regenerative therapy. Initial gingival thickness has been reported to be the most significant factor influencing the prognosis of a complete root coverage procedure. Gingival biotypes therefore appear to be decisive in the success of periodontal treatment. Therefore, in this systematic review, we aimed to investigate the relationship between the gingival biotype with surgical procedures results for root coverage. The review question was: What are the outcomes in different gingival biotypes following root coverage procedure?

## Material and Methods

This systematic review was based on the Cochrane Manual of systematic reviews of interventions (11) following the PRISMA recommendations(12) and was registered in the International Register for Systematic Reviews (PROSPERO) with the following identification number: CRD42020209570.

-PICO question

For patients with different gingival biotypes, what are the outcomes when root coverage procedures are followed?

-Literature Search Strategy

Two reviewers independently evaluated the titles and abstracts in the first phase of screening and the full-text articles in the second phase. The screening processes were conducted between August 31, 2010 and August 31, 2020. Electronic searches of the National Library of Medicine (PubMed MEDLINE), EBSCO, Science Direct, EMBASE and Cochrane were performed. An additional search for unpublished data in the gray literature was carried out. Finally, language restrictions were established considering only publications in English and Spanish. Relevant articles were searched using the following key terms and Boolean operators (AND, OR, NOT): (“gingival recession”[MeSH Terms] OR (“gingival”[All Fields] AND “recession”[All Fields]) OR “gingival recession”[All Fields]) AND (“biotype”[All Fields] OR “biotypes”[All Fields] OR “biotypic”[All Fields]) (“gingival recession”[MeSH Terms] OR (“gingival”[All Fields] AND “recession”[All Fields]) OR “gingival recession”[All Fields]) AND (“biotype”[All Fields] OR “biotypes”[All Fields] OR “biotypic”[All Fields]).

-Inclusion Criteria

The inclusion criteria included: 1) Randomized or non-randomized clinical trials and observational studies, 2) The presence of single or multiple gingival recessions, 3) Patients older than 18 years, systemically healthy or controlled adults, 4) Surgery intervention of “root covering procedure in subjects with different gingival biotypes.” 5) Recessions around the teeth could have been treated with several approaches (coronal displacement flap (CAF) surgical procedure, tunneling, or pedicle flap) described in the literature (11). 6) The objective of root covering procedures should be to completely cover the exposed root surface(s) of the affected tooth/teeth. 7) Study should have made explicit the results of the different surgical techniques for root coverage in a thin biotype compared to a thick biotype.

The final result of the different surgical techniques for root coverage according to the gingival biotype was evaluated by the Aesthetic Root Coverage Score (RES) (9). For RES, the main gingival indicator is root coverage, which is considered successful when it reaches 60%. Regarding the evaluation of the final position of the gingival margin; three points are awarded for partial root coverage and six points for complete root coverage; zero points are assigned when the final position of the gingival margin is equal to the initial or apical position of the previous recession. In this case, zero points is equal to 0%, three points are 50%, and six points are 100%, a perfect score. Patient satisfaction with the procedure and the RES variables: marginal tissue contour, soft tissue texture, alignment of the mucogingival junction, and gingival color were also taken into account as part of the results.

-Exclusion criteria

Pre-clinical studies, animal studies, repeated reports from the same study or author, studies that reported results of root coating surgeries considering only the thickness of the mucosa without differentiating the biotype.

-Data extraction and collection

**Figure 1:**
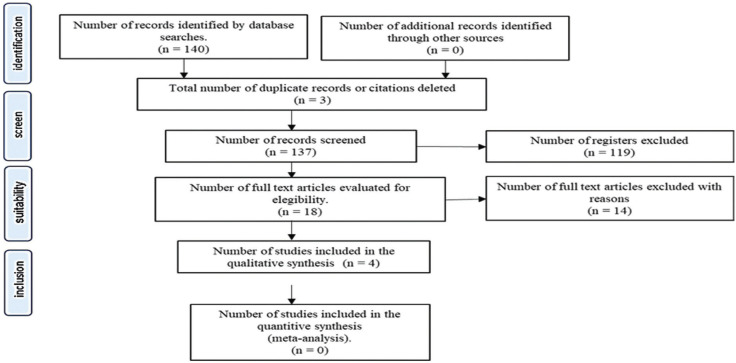
Flow chart of the study selection.

Data on the root coverage esthetic score (RES) and its five components (gingival margin level, marginal tissue contour, soft tissue texture, mucogingival junction alignment, and gingival color) were extracted. In addition, morbidity and patient satisfaction (measured as visual analog score; VAS) were also extracted. The flow chart (PRISMA) (Fig. 1) shows the study selection process.

The selected non-randomized clinical trials were independently assessed by two reviewers according to the Methodological Index for Non-Randomized Studies (MINORS) (13) .

•Strategy for Data Synthesis

Comparisons in pairs were made for general results. All the results were explained initially in a descriptive way. Finally, the factors related to the patients and the surgery factors that affect the results were analyzed.

•Analysis of Subgroups

All the clinical variables related with the patient were explored with respect to their effect on gingival biotype after the procedures. Confounding variables were found.

## Results

-Search

The selection process from MEDLINE, Science Direct, EMBASE and Cochrane and additional sources subjected to manual search yielded 140 articles, as reported in the flowchart (Fig. 1).

A total of 137 studies were included after abstract screening, and 18 reports were evaluated for eligibility, finally only four studies accomplished the inclusion criteria. The four classified studies considered procedures for surgical root coverage of 409 recessions (Table 1). These studies accurately reported root coverage after the surgery, but only Kim *et al*. (14) and Rasperini *et al*. (15) registered the RES index (Table 2). The complete root coverage was reported in four studies, between 70% to 86.12% patients with thin biotype and between 77.8% to 96% in patients with thick biotype. According to the results, the evaluation of gingival biotype could be served as an additional parameter for decision making on the best surgical approach.

**Table 1:**
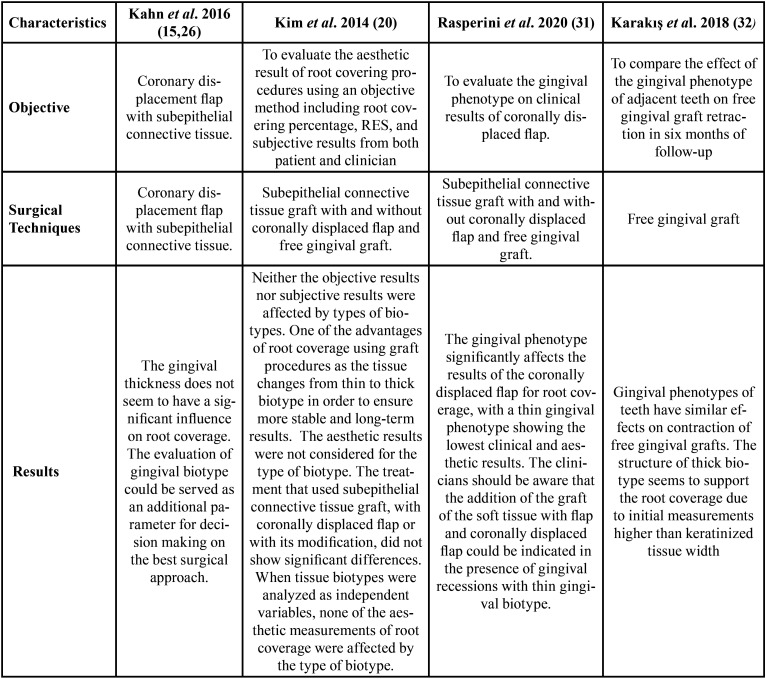
Detailed description of selected studies.

**Table 2:**
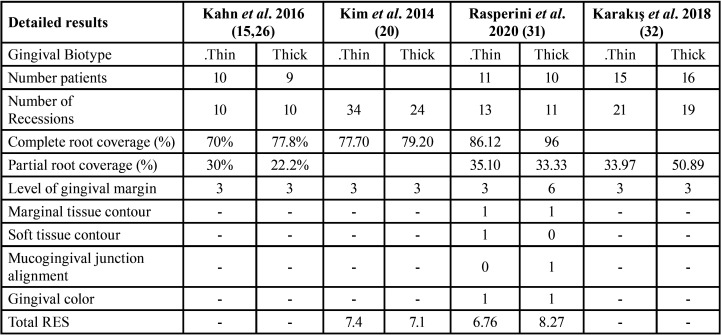
Detailed results of selected studies.

The structure of thick biotype seems to support the root coverage due to initial measurements of keratinized tissue width. The gingival thickness does not seem to have a significant influence on root coverage.

One of the advantages of root coverage using graft procedures is the change of tissue from thin to thick biotype to ensure more stable and long-term results. The aesthetic results were not considered for the initial classification of the biotype. The treatment that used subepithelial connective tissue graft, with coronally displaced flap or with its modification, did not show significant differences. When gingival biotypes were analyzed as independent variables, none of the aesthetic measurements of root coverage were affected by the class of biotype.

The selected studies considered that the addition of the graft of connective tissue with flap and coronally displaced flap could be indicated in the presence of gingival recessions with thin gingival biotype. The partial root coverage was reported as a positive result for patients and dentists in case of intervention of severe gingival recessions. According to the studies considered, the aesthetic result of the procedures of root coverage should be performed through a comprehensive and non-particular approach. (Table 2). The comprehensive approach is related to clinical, systemic conditions and patient age.

**Figure 2:**
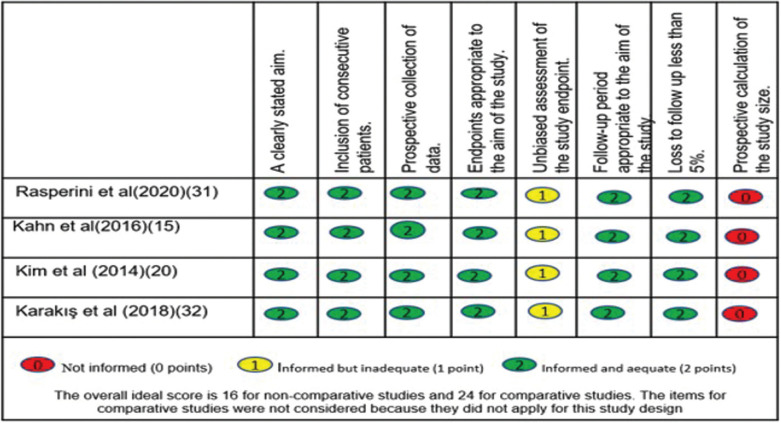
Evaluation of biases according to the MINORS scale.

In the evaluation of biases, the methodological index for non-randomized studies (MINORS)(13) was considered. The studies recorded as “Not informed” the item corresponding to the prospective study size calculation. The criteria corresponding to comparative studies were not applied since none of them fulfilled it in their methodology. All had a score of “one” on the item “Impartial evaluation of the study endpoint.” All studies had the same final score of 13/16 corresponding to a bias of 18.75%, which is considered low (Fig. 2).

## Discussion

Gingival thickness is considered an important parameter in achieving complete root coverage. A gingival thickness of 0.8 - 1.2 mm is associated with a more predictable prognosis(16). The articles selected in this SR report a significant positive correlation between the gingival biotype and the recession defect generated. This suggests that the tissue biotype is a significant factor that influences the results of the aesthetic treatment of the root coverage (17). Finally, the initial gingival thickness was the most influential factor and the gingival biotype greater than or equal to 1.2 mm was associated with complete root coverage (18).

Previous studies have reported unfavorable results at sites with width ≥3 mm and depth in recessions ≥5 mm (19). The level of interdental periodontal support has proven to be an important factor for the result of root coverage procedures and the epithelialized free gingival tissue graft results in a lower percentage of root coverage when compared to the connective tissue graft (20).

The expected successful end result after gingival recession treatment is root coverage. Commonly, the achievement of such a result will allow not only the aesthetic correction but also the functional solution, for example, the resolution or reduction of thermal and touch hypersensitivity or the prevention of root abrasion (21). It is stated that the greater the initial recession, the less the opportunity to achieve complete root coverage (9). Rasperini *et al*. (22) assert that the gingival biotype does not affect the results of the coronally displaced flap when using autologous connective tissue graft, suggesting that this approach should be recommended only in the case of a thin gingival phenotype (23). However, it was shown that the coronally displaced flap with an autologous connective tissue graft provides superior results to a coronally displaced flap when the gingival thickness is ≤ 0.8 mm. This leads Cairo *et al*. to conclude that the coronally displaced flap surgical procedure, including autologous connective tissue graft, has additional benefits only in thin gingival biotypes (24,25).

The studies considered in this SR show strengths. All patients were treated by trained periodontists and patient data were recorded using standardized questionnaires and qualitative methods. The main limitations of the studies selected in this review were lack of randomization of patients, strict inclusion criteria, lack of assessment of postoperative pain and color matching of grafted areas, as well as small sample size. These limitations are related to the absence of representativeness, the impossibility of making statistical statements about the results and the risk of incurring bias due to the sampling criteria used. The blinding of the participants also could not be met due to the ethical impossibility of hiding the kind of surgery to be performed or the surgical options that existed. It should be noted that in none of the studies was the condition of the blind evaluation of the results explicit, that is to say that the periodontal status before and after surgery was assessed by a periodontist who was not involved in the investigation and was unaware of its objectives. It must be positively recognized that when applying the MINORS bias scale, the four selected studies had a rating of 13/16, which indicates low bias.

The results of this SR cannot be generalized to other populations, due to strict inclusion criteria and lack of randomization. It is not possible to definitively recommend any of the tested procedures, since there could be subjectivity in the assessment by the evaluators. According to the studies in this SR, patients should be informed that they could experience greater postoperative pain and discomfort sensations, greater gingival coverage (18%) and a better aesthetic result when using autologous grafts.

The articles that compare the results of surgical procedures for the solution of gingival recessions according to the gingival biotype are scarce and all are classified in non-randomized clinical trials. Most studies only report how much tissue thickness increases after the procedure without specifically relating it to the gingival biotype.

When tissue biotypes are analyzed as independent variables in the four selected studies, none of the root covering procedures was affected by the biotype class.
